# Effects of Tofacitinib on Muscle Remodeling in Experimental Rheumatoid Sarcopenia

**DOI:** 10.3390/ijms241713181

**Published:** 2023-08-24

**Authors:** Ismael Bermejo-Álvarez, Sandra Pérez-Baos, Paula Gratal, Juan Pablo Medina, Raquel Largo, Gabriel Herrero-Beaumont, Aránzazu Mediero

**Affiliations:** Bone and Joint Research Unit, Rheumatology Department, IIS-Fundación Jiménez Díaz UAM, 28040 Madrid, Spain

**Keywords:** rheumatoid arthritis, sarcopenia, TOFA, JAK inhibitors, CK, cytokines, muscle regeneration, intracellular pathways, treatment, animal model

## Abstract

Sarcopenia is a frequent comorbidity of rheumatoid arthritis (RA). Clinical trials have shown that JAK inhibitors (JAKi) produce an asymptomatic increase in serum creatine kinase (CK) in RA, suggesting an impact on muscle. We evaluated the effect of JAKi in muscle remodeling in an experimental RA model. Antigen-induced arthritis (experimental RA, e-RA) was performed in 14 rabbits. Seven rabbits received tofacitinib (TOFA, orally 10 mg/kg/day). Animals were euthanized one day after the last ovalbumin injection, and muscles were prepared for histology, RT-PCR, and WB. C-reactive protein (CRP) and Myostatin (MSTN) serum concentration were determined by ELISA. Creatine and creatine kinase (CK) were analyzed. An increase in body weight as well as tibialis anterior cross-sectional area and diameter was observed in e-RA+TOFA vs. e-RA. e-RA decreased type II fibers and increased the myonuclei number, with all reverted by TOFA. TOFA did not modify CRP levels, neither did MSTN. TOFA significantly reduced IL-6, atrogin-1, and MuRF-1 compared with e-RA. e-RA+TOFA showed higher CK and lower creatine levels compared with e-RA. No differences in PAX-7 were found, while TOFA prevented the increase in MyoD1 in e-RA. Our model reflects the features of rheumatoid sarcopenia in RA. JAKi increased muscle mass through attenuating IL-6/JAK/STAT activation, decreasing atrogenes, and restoring muscle differentiation markers. These data together with an increase in CK support the role of CK as a valuable marker of muscle gain following JAKi treatment.

## 1. Introduction

In addition to joint damage, rheumatoid arthritis (RA) confers a higher risk of several comorbidities including cardiovascular disease and infections [1]. Alterations in body composition including reduced fat-free mass, primarily in the form of muscle mass, and stable or increased fat mass may result in minimal changes in body mass index [2]. This condition is known as rheumatoid sarcopenia (RS). Loss of body cell mass is independent of energy intake, therapy, or duration of disease [3]. A recent meta-analysis showed that RS prevalence varies between 1% and 54%. This variation may be due to the lack of consensus on the clinical criteria for diagnosis and the heterogeneity of the RA populations studied. In any case, its presence correlates with a high risk of physical disability, morbidity, and mortality [4].

Muscle turnover is maintained through an accurate balance between pro/anti-anabolic and anti-catabolic factors. A shift in this balance towards inflammatory mediated catabolism appears to be the key factor in RS [5,6]. Unfortunately, current therapies fail in reverting RS despite achieving RA remission. The hypercatabolic status of RA patients leads to a ‘permanent footprint’ that persists even after controlling disease activity [7].

Cytokine-mediated catabolism represents the major pathogenic factor of RS [8]. A complex network of these peptides and its downstream cascades, such as the janus kinase/signal transducer and activator of transcription (JAK/STAT) pathway, have emerged to have a key role during muscle development and regeneration [9,10,11,12]. Pro-inflammatory myokines can also be involved in the crosstalk between skeletal muscle and other organs, as IL-6 produced by contracting skeletal muscle regulates metabolism in other organs [9,13,14]. Therefore, a lack of physical activity affects a whole network of organs including liver, pancreas, fat, and bone. These opposite outcomes may rely on the fact that these signaling cascades are involved in both homeostasis and inflammation, and this is the case of the JAK/STAT pathway. Different members of the JAK/STAT family differentially regulate cell proliferation and differentiation [13,15]. This fact highlights the relevance of studying these downstream elements when assessing the effects of inflammation and its blockade on muscle homeostasis.

Previously, we have demonstrated that antigen-induced arthritis closely mirrored human RA, including an RS with increased muscle protein breakdown and a compensatory anabolic response [16]. The diseased animals had a reduction in weight and muscle size and an up-regulation of atrogin-1 and muscle RING-finger protein-1 (MuRF-1) in muscle. Strikingly, there was a paradoxical decrease in Myostatin expression, along with a reduction in phosphorylated signal transducer and activator of transcription 3 (pSTAT3) levels, which pointed to the existence of a compensatory anabolic activation. Creatine kinase (CK) is found in both the cytosol and mitochondria of tissues where energy demands are high, mainly in muscle [17]. CK catalyzes the reversible phosphorylation of creatine to phosphocreatine, and it is in charge of buffering ATP levels and transport from mitochondria to sites of ATP consumption in the cytosol [17,18]. This shuttle system is critical for the production and maintenance of energy supply and is involved in the metabolic feedback regulation of respiration [19]. Elevations in CK are indicative of neuromuscular conditions including myopathy and rhabdomyolysis [20], as well as exercise, endocrine disorders, and several drugs and supplements [21]. Unexpectedly, JAK inhibitors (tofacitinib (TOFA), baricitinib, and upadacitinib) induce increased CK serum levels in patients with inflammatory disorders [22,23]. While CK increases can be indicative of muscle damage, there are no other indicators of muscle pathology observed with JAK inhibitors [24]. In the case of tofacitinib, CK elevations have been shown to be reversible after withdrawal in psoriatic patients as well as ulcerative colitis and RA [22,25,26].

The aim of this study is to investigate the early and direct consequences of JAK/STAT inhibition in a rabbit model of RA (experimental RA, e-RA). Moreover, we have evaluated both circulating and muscular CK levels in our model to understand if they mimic the elevation found in patients treated with JAK inhibitors and whether this blockade exerts a beneficial effect.

## 2. Results

### 2.1. Weight and CRP

**Figure 1:**
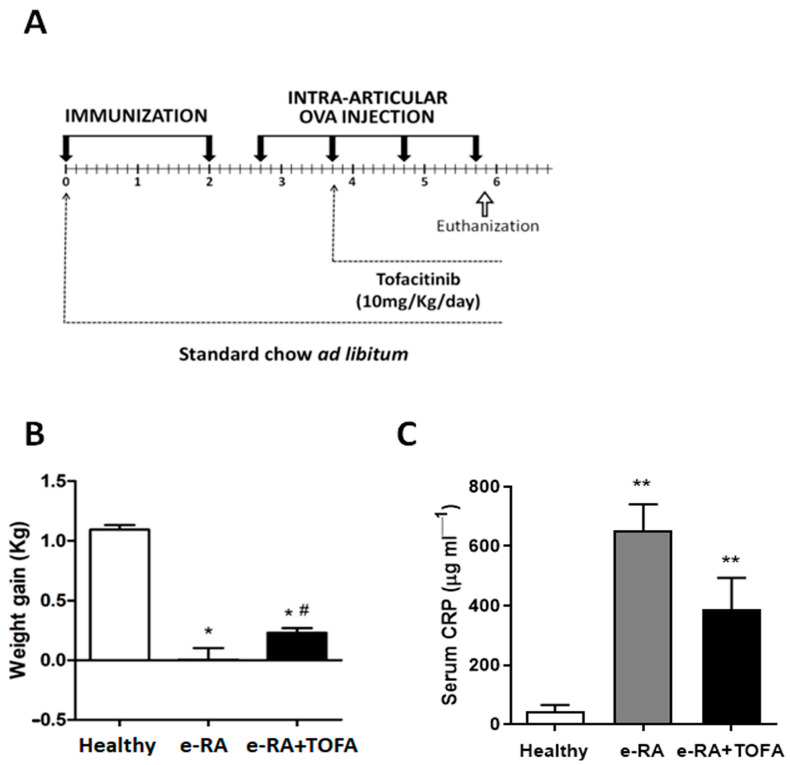
Experimental model of rheumatoid arthritis in rabbits that replicate human disease. (**A**) Chronogram of the experimental model. (**B**) Body weight gain (kg) of rabbits at the end of the study. (**C**) Serum CRP levels (μg/mL) at the end of the study. Data are shown as the mean and SEM (*n* = 7 rabbits per group). * *p* < 0.05 and ** *p* < 0.01 vs. healthy # *p* < 0.05 vs. CRP: C-reactive protein; e-RA: experimental rheumatoid arthritis; OVA: ovalbumin; TOFA: tofacitinib.

As described before [16], the induction of e-RA showed a reduction in rabbit weight that was significantly reverted by TOFA (tofacitinib) (Figure 1B). When C-reactive protein (CRP) levels were analyzed, we also observed that e-RA rabbits showed higher serum CRP levels compared with the healthy group (652.14 ± 88.78 μg/mL vs. 44.55 ± 21.56 μg/mL healthy, *p* = 0.0012) (Figure 1C), and TOFA treatment led to a downwards trend in circulating CRP levels (388.71 ± 104.9 μg/mL vs. 652.14 ± 88.78 μg/mL e-RA, *p* = ns) (Figure 1C).

### 2.2. Structural and Histological Changes in Skeletal Muscle

**Figure 2:**
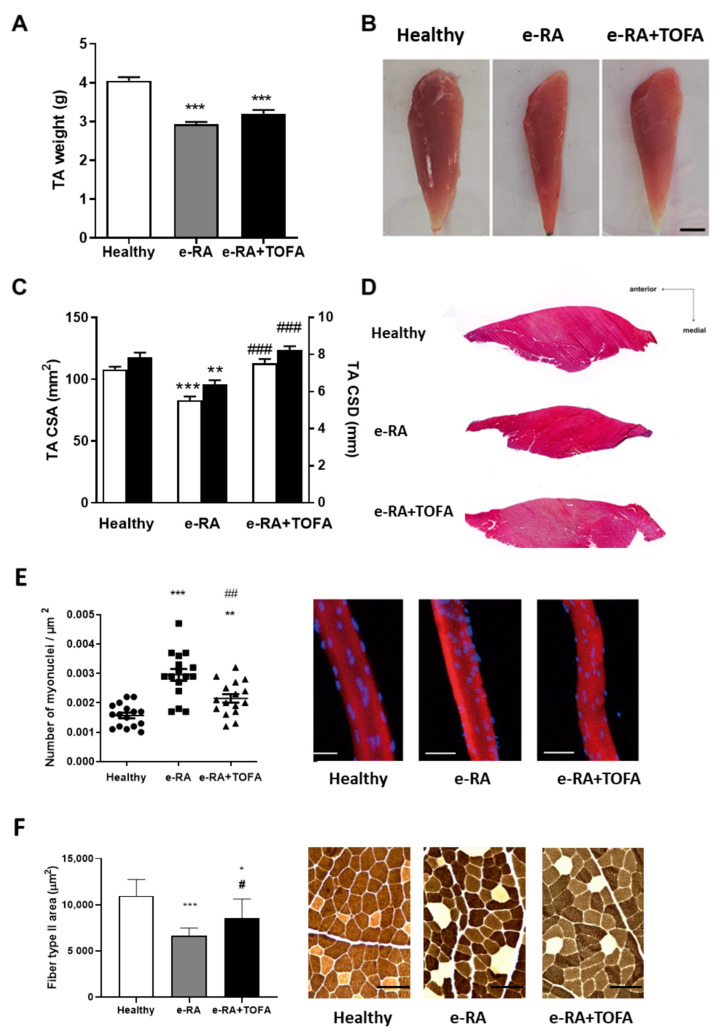
TOFA improves structural skeletal muscle changes. (**A**) Tibialis anterior (TA) weight (g). (**B**) Representative TA from healthy control and e-RA groups. Posterior view, scale bar = 1 cm. (**C**) Cross-sectional diameter of TA (mm) and cross-sectional area of TA (mm^2^). (**D**) Representative mid-belly cross sections of TA in control and e-RA groups stained with H&E. Scale bar = 2.5 mm. (**E**) Representative focal microscopy images from segments of extensor digitorium longus (EDL) fibers in healthy and e-RA groups. Rhodamine phalloidin (red) was used to stain actin and DAPI (blue) to stain nuclei. Scale bar = 30 μm. Number of myonuclei in healthy in comparison with e-RA groups expressed as number of nuclei per 100 mm segment. (**F**) Representative sections of type I (white) and II (black) fiber distribution and size in TA of healthy and e-RA groups stained with ATPase pH 9.4. Scale bar = 100 μm. Data are shown as the mean and SEM (*n* = 7 rabbits per group). * *p* < 0.05, ** *p* < 0.01, and *** *p* < 0.001 vs. healthy; # *p* < 0.05, ## *p* < 0.01, and ### *p* < 0.001 vs. e-RA. EDL: extensor digitorum longus; e-RA: experimental rheumatoid arthritis; TA: tibialis anterior; TOFA: tofacitinib.

To analyze structural changes in tibialis anterior (TA), we addressed muscle weight. TA weight was diminished in e-RA rabbits compared with healthy groups (2.93 ± 0.09 g vs. 4.05 ± 0.06 g healthy, *p* < 0.001) and tended to increase with TOFA, albeit statistical significance was not found (3.2 ± 0.09 g vs. 2.93 ± 0.09 g e-RA, *p* = 0.069) (Figure 2A). TA muscle size, which diminished in e-RA, was significantly improved by TOFA, as demonstrated in both anterior macroscopic photographs (Figure 2B). Measurement of H&E sections showed reduced TA cross-sectional diameter (CSD) and cross-sectional area (CSA) in e-RA compared with healthy groups (82.94 ± 3.16 mm^2^ vs. 107.9 ± 2.23 mm^2^ healthy CSA and 7.85 ± 0.26 mm vs. 6.39 ± 0.22 mm healthy CSD, *p* < 0.001 and *p* = 0.0013, respectively) (Figure 2C,D), and TOFA reverted these parameters (112.9 ± 3.55 mm^2^ vs. 82.94 ± 3.16 mm^2^ e-RA CSA and 8.24 ± 0.21 mm vs. 6.39 ± 0.22 mm e-RA CSD, both *p* < 0.001) (Figure 2C,D).

We next analyzed the shape and different spatial organization of nuclei in the fiber. We found an increase in extensor digitorum longus (EDL) myonuclear number and disorganization in e-RA compared with healthy groups (0.0029 ± 0.002 myonuclei/μm^2^ vs. 0.0015 ± 9 × 10^−5^ myonuclei/μm^2^ healthy, *p* < 0.001), and TOFA restored the myonuclei number to healthy levels (0.0021 ± 0.00014 myonuclei/μm^2^ vs. 0.0029 ± 0.002 myonuclei/μm^2^ e-RA, *p* < 0.01), improving myonuclear organization, along with an apparent decrease in nuclei size (Figure 2E).

ATPase staining showed a reduction in type II fiber area in e-RA when compared with healthy groups (6636 ± 265.4 μm^2^ e-RA vs. 10,964 ± 592.6 μm^2^ healthy, *p* < 0.001), which was significantly increased by TOFA (8564 ± 650.8 μm^2^ e-RA+TOFA vs. 6636 ± 265.4 μm^2^ e-RA, *p* < 0.05) (Figure 2F).

### 2.3. Myogenic Markers in Skeletal Muscle

**Figure 3:**
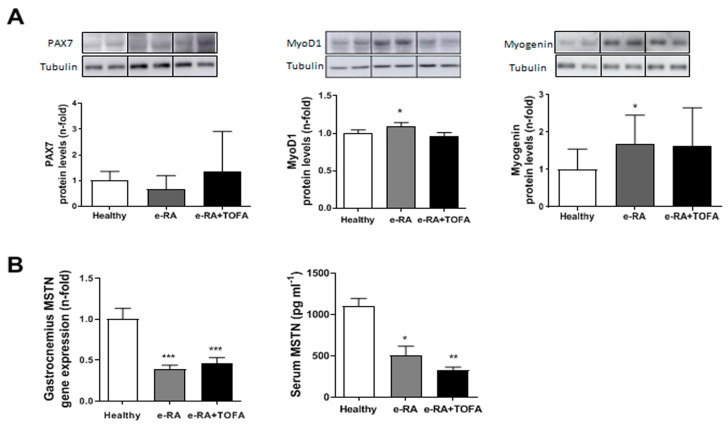
No differences in PAX-7 and MSTN were found, while TOFA tended to revert MyoD1 expression. (**A**) Densitometric analysis of Pax7, MyoD1, and Myogenin protein expression levels in gastrocnemius. Data are normalized to endogenous control (α-tubulin) and expressed as arbitrary units (A.U.). Representative cropped blots of two animals of each group are shown: healthy, e-RA, and e-RA+TOFA, respectively. (**B**) Gene and serum expression of MSTN. Data are shown as the mean and SEM (*n* = 7 rabbits per group). * *p* < 0.05, ** *p* < 0.01, and *** *p* < 0.001 vs. healthy. e-RA: experimental rheumatoid arthritis; MSTN: Myostatin; TOFA: tofacitinib.

Next, we assessed levels of the myogenic markers Pax7, MyoD1, and Myogenin in gastrocnemius. No chances in Pax7 were found among groups (0.67 ± 0.14 e-RA vs. 1.36 ± 0.43 e-RA+TOFA vs. 0.99 ± 0.09 healthy, *p* = ns) (Figure 3A). We found increased protein levels of MyoD1 in e-RA (1.09 ± 0.05 e-RA vs. 1 ± 0.04 healthy, *p* < 0.05) and a downward trend after TOFA (0.96 ± 0.05 vs. 1.09 ± 0.05 e-RA, *p* = ns) (Figure 3A). We observed a significant increase in Myogenin levels in e-RA when compared with healthy groups (1.68 ± 0.21 e-RA vs. 0.99 ± 0.15 healthy, *p* < 0.05) and, surprisingly, TOFA also tended to increase Myogenin (1.62 ± 0.29 vs. 1.68 ± 0.21 e-RA, *p* = ns) (Figure 3A).

When we assessed the levels of Myostatin (MSTN) in gastrocnemius and serum, we observed decreased levels of this anti-anabolic factor in e-RA, both locally (0.39 ± 0.05 e-RA vs. 1 ± 0.13 healthy, *p* < 0.0001) and systemically (503.7 ± 114.3 e-RA vs. 1103 ± 93.62 healthy, *p* < 0.05) (Figure 3B). No changes were found with TOFA, neither locally (0.46 ± 0.07 vs. 0.39 ± 0.05 e-RA, *p* = ns) nor systemically (326.7 ± 37.11 vs. 503.7 ± 114.3 e-RA, *p* = ns) (Figure 3B).

### 2.4. Atrogenes and Proinflammatory Mediators in Skeletal Muscle

**Figure 4:**
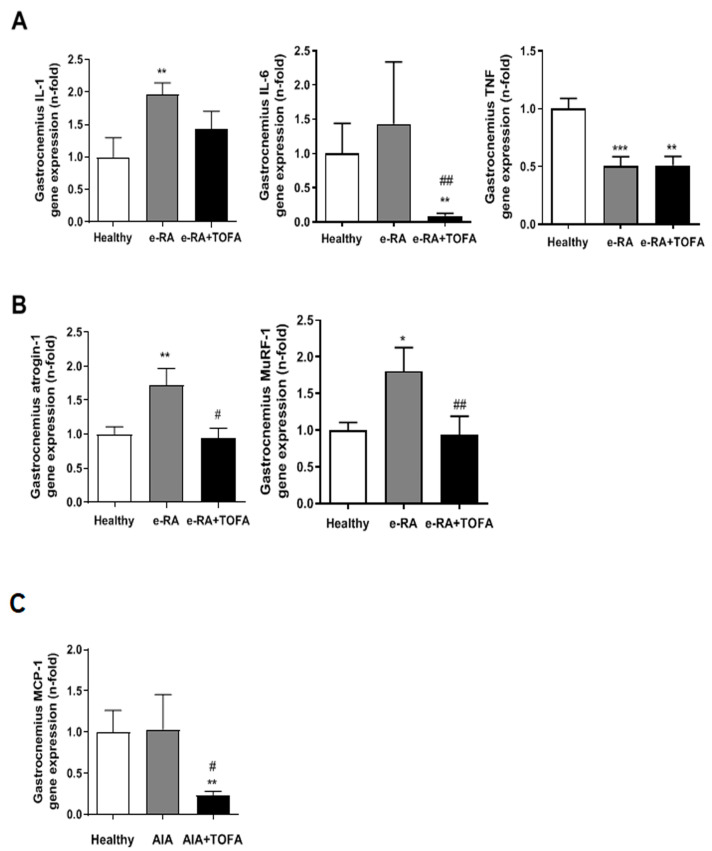
Pro-inflammatory mediators and atrogenes. (**A**) Gene expression for IL-1β, IL-6, and TNFα. (**B**) Gene expression for atrogin-1 and MuRF1. (**C**) Gene expression for MCP-1. Data are shown as the mean and SEM (*n* = 7 rabbits per group). * *p* < 0.05, ** *p* < 0.01, and *** *p* < 0.001 vs. healthy; # *p* < 0.05 and ## *p* < 0.01 vs. e-RA. e-RA: experimental rheumatoid arthritis; IL-1: interleukin 1β; MCP-1: monocyte chemotactic protein-1; MuRF1: muscle RING-finger protein-1; TOFA: tofacitinib; TNF: tumor necrosis factor.

When we analyzed if the pro-inflammatory mediator and atrogenes were modulated, we observed that the arthritic insult led to an increase in IL-1β mRNA levels (1.96 ± 0.18 vs. 0.99 ± 0.3 healthy, *p* < 0.01), and TOFA tended to restore the values (1.44 ± 0.27 vs. 1.96 ± 0.18 e-RA, *p* = ns) (Figure 4A). On the other hand, IL-6 gene expression was not modified in e-RA as compared with healthy groups (1.43 ± 0.9 vs. 1 ± 0.4 healthy, *p* = ns), but TOFA exerted a decrease in mRNA levels of this cytokine (0.09 ± 0.038 vs. 1.43 ± 0.9 e-RA, *p* < 0.01) (Figure 4A). TNFα gene expression was diminished in both e-RA and e-RA+TOFA groups compared with healthy groups (0.5 ± 0.08 e-RA vs. 0.51 ± 0.08 e-RA+TOFA vs. 1 ± 0.09 healthy, *p* < 0.001 and *p* < 0.01, respectively, vs. healthy) (Figure 4A).

When atrogenes were analyzed, e-RA animals exhibited an upregulation of both atrogin-1 (1.72 ± 0.25 vs. 1 ± 0.11 healthy, *p* < 0.01) and MuRF-1 (1.80 ± 0.32 vs. 1 ± 0.10 healthy, *p* < 0.05), which was completely restored with TOFA (0.94 ± 0.15 vs. 1.72 ± 0.25 e-RA atrogin-1, *p* < 0.05, and 0.93 ± 0.26 vs. 1.80 ± 0.26 e-RA MuRF-1, *p* < 0.01) (Figure 4B).

Finally, monocyte chemotactic protein-1 (MCP-1) gene expression was not modified in e-RA (1.03 ± 0.42 vs. 1 ± 0.26 healthy, *p* = ns), but it was significantly decreased with TOFA (0.24 ± 0.4 vs. 1 ± 0.26 healthy, *p* < 0.01) (Figure 4C).

### 2.5. Creatine Kinase, Creatine, and Pyruvate Activation

**Figure 5:**
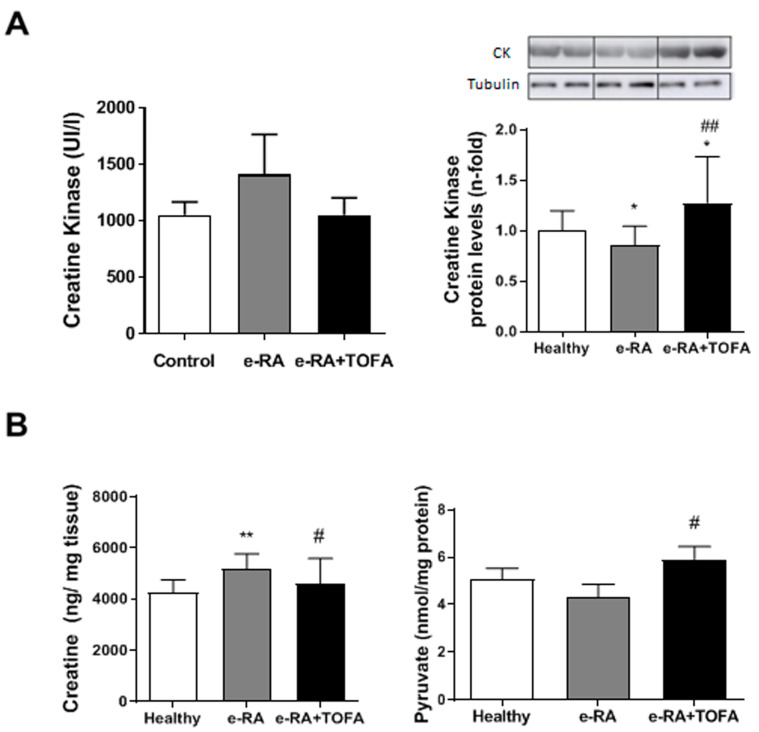
TOFA showed increased creatine kinase and lower creatine in muscle. (**A**) Serum and tissue CK levels. (**B**) Creatine (ng/mg tissue) and pyruvate (nmol/mg protein) in muscle. Data are shown as the mean and SEM (*n* = 7 rabbits per group). * *p* < 0.05 and ** *p* < 0.01 vs. healthy; # *p* < 0.05 and ## *p* < 0.01 vs. e-RA. CK: creatine kinase; e-RA: experimental rheumatoid arthritis; TOFA: tofacitinib.

To confirm the increase in serum CK and creatine levels observed in RA patients under JAK inhibitors, we analyzed CK in both serum and gastrocnemius and creatine in gastrocnemius. Serum CK levels were not altered in any group (1049 ± 116.5 healthy vs. 1410 ± 353.9 e-RA vs. 1049 ± 153.9 e-RA+TOFA, *p* = ns) (Figure 5A). Nevertheless, e-RA induced a significant reduction in muscle CK when compared with healthy groups (0.86 ± 0.05 vs. 0.99 ± 0.05 healthy, *p* < 0.05), and TOFA increased CK levels (1.28 ± 0.13 vs. 0.86 ± 0.05 e-RA, *p* < 0.01) (Figure 5A). We observed an increase in creatine in e-RA (5155 ± 213.2 ng/mg tissue vs. 4237 ± 176.4 ng/mg tissue healthy, *p* < 0.01), which was normalized by TOFA (4570 ± 380.3 ng/mg tissue vs. 5155 ± 213.2 ng/mg tissue e-RA, *p* < 0.05) (Figure 5B). Finally, pyruvate concentrations were analyzed as a marker of the muscle metabolic activity. e-RA induced a non-significant reduction in pyruvate when compared with healthy groups (4.29 ± 0.56 nmol/mg protein vs. 5.07 ± 0.46 nmol/mg protein healthy, *p* = ns), which was reverted by TOFA (5.91 ± 0.54 nmol/mg protein vs. 4.29 ± 0.56 nmol/mg protein e-RA, *p* < 0.05) (Figure 5B).

### 2.6. Levels of pSTAT1, pSTAT3, SOCS1, and SOCS3 in Skeletal Muscle

**Figure 6:**
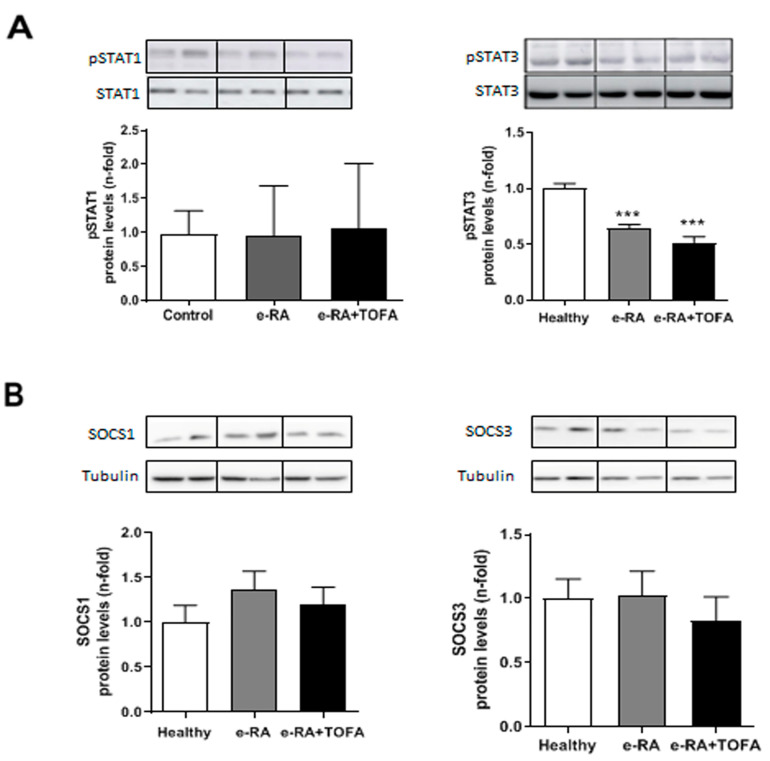
TOFA decreases STAT3 but not STAT1 signaling. (**A**) Densitometric analysis of pSTAT1 and pSTAT3 protein expression in gastrocnemius. (**B**) Densitometric analysis of SOCS1 and SOCS3 protein expression in gastrocnemius. Data are normalized to endogenous control (α-tubulin) and expressed as arbitrary units (A.U.). Representative cropped blots of two animals of each group are shown: healthy, e-RA, and e-RA+TOFA, respectively. Data are shown as the mean and SEM (*n* = 7 rabbits per group). *** *p* < 0.001 vs. healthy. e-RA: experimental rheumatoid arthritis; TOFA: tofacitinib.

To determine the precise contribution of the JAK/STAT pathway to the muscle response, we explored levels of pSTAT1, pSTAT3, and suppressor of cytokine signaling 1 and 3 (SOCS1 and SOCS3) in gastrocnemius. pSTAT1 was not modified in either e-RA or e-RA+TOFA when compared with healthy groups (0.94 ± 0.2 e-RA vs. 1.07 ± 0.26 e-RA+TOFA vs. 0.97 ± 0.09 healthy, *p* = ns) (Figure 6A), while pSTAT3 fell both in e-RA and e-RA+TOFA (0.64 ± 0.03 e-RA vs. 0.52 ± 0.05 e-RA+TOFA vs. 1 ± 0.05 healthy, *p* < 0.001) (Figure 6A). We did not find changes between groups in either SOCS1 (1.36 ± 0.21 e-RA vs. 1.19 ± 0.2e-RA+TOFA vs. 17 ± 0.19 healthy, *p* = ns) or SOCS3 (1.02 ± 0.2 e-RA vs. 0.82 ± 0.19 e-RA+TOFA vs. 1 ± 0.15 healthy, *p* = ns) (Figure 6B).

## 3. Discussion

Despite having a severe systemic inflammatory state, JAK inhibition was able to rapidly increase muscle mass by attenuating the activation of IL-6/JAK/STAT, reducing atrogene expression and restoring muscle cell differentiation markers in the tissue back to baseline levels. The effect was accompanied by an increase in muscle CK, which supports its usefulness as a marker for muscle gain following treatment with JAK inhibitors. Notably, the direct effect of JAK inhibition on muscle remodeling occurred in an environment with high levels of inflammation. e-RA rabbits that had received TOFA 10 mg/Kg/day for two weeks were sacrificed one day after the fourth and last i.a. ovalbumin (OVA) injection (Figure 1A). Therefore, the treatment did not aim to fully control the inflammatory response, but rather to understand the direct effect of TOFA on muscle remodeling in RS.

RA increases the risk of several comorbidities, including RS [2]. Our animal model replicated both RA and RS, consistent with other models of inflammatory arthritis [27,28]. e-RA rabbits exhibited reduced muscle CSA, CSD and weight, heterogeneous myofiber size, and loss of type II fibers, as occurs in other species [29]. Treatment with TOFA reversed these features, but did not restore the decreased levels of muscle and serum MSTN. MSTN negatively regulates muscle homeostasis by inhibiting protein synthesis via inhibition of the PI3K/Akt/mTOR pathway and activating catabolism via the ubiquitin-proteasome system [30,31]. The decrease in MSTN facilitates muscle anabolism in e-RA, which is maintained in the presence of TOFA to compensate for muscle mass loss [32,33].

Another key molecule in skeletal muscle is MCP-1 and it role in macrophage recruitment [34]. An overexpression in MCP-1 induced a decrease in muscle weight due to the metabolic alterations as well as mTOR inhibition [35]. The marked decrease in MCP-1 gene expression we observed in the gastrocnemius in TOFA-treated animals leads us to anticipate a decrease in the number of macrophages infiltrating the muscle and, therefore, a decrease in the specific inflammation caused by MCP-1 and a reestablishment of anabolic pathways essential for protein synthesis.

Overall, it appears that TOFA has a positive remodeling effect by counteracting the muscular atrophy induced by inflammation in arthritis. Similar results were observed in a dystrophic murine model. Despite the intense systemic inflammation, TOFA decreased the expression of inflammatory myokines and atrogenes, as well as the activity of the ubiquitin-proteasome system. These results suggest that the effect of TOFA is mediated by JAK/STAT regulation in myocytes.

Adult skeletal muscle can respond to environmental stimuli and secrete trophic factors, such as IL-6, which is recognized as myokines. In our model, we observed an increase in IL-6 mRNA expression in e-RA animals, which was completely abolished when rabbits were treated with TOFA. As is well established, IL-6 is involved in hypertrophy [36]; it is induced by resistance exercise in human muscle, in electrically stimulated cultured human myotubes [37,38], and in control satellite cell proliferation [36]. IL-6 signals through the gp130 receptor, with the JAK/STAT pathway being the main intracellular effector. This is consistent with the dual functions of IL-6 in myogenesis, promoting both myoblast proliferation and/or differentiation.

JAK-mediated pathways play a key role during development and are important in muscle atrophy and regeneration [9,10,11,12]. The importance of the JAK/STAT pathway is well established in skeletal muscle diseases that promote muscle wasting, such as muscular dystrophy and cancer cachexia [39]. Different combinations of the JAK/STAT pathway can have opposite effects on muscle differentiation and myogenesis [13,15,40]. The JAK1/STAT1/STAT3 pathway is an important regulator of myoblast proliferation and differentiation, working as a checkpoint to ensure that differentiation does not start prematurely [15]. This pathway promotes myoblast proliferation by blocking the expression of myoblast differentiation and fusion markers, such as MyoD1, MEF2, and Myogenin, while promoting the expression of genes involved in cell cycle progression and proliferation. STAT3 is able to directly associate with MyoD1 and inhibit its myogenic activities when overexpressed in C2C12 cells [41]. On the other hand, Wu et al., showed that JAK2 is required for myogenic differentiation through the activation of STAT2 and STAT3. Specifically, they found that JAK2 inhibition led to a decrease in Myogenin expression and myotube formation, while JAK2 overexpression enhanced myogenic differentiation in vitro. The authors suggest that JAK2/STAT2/STAT3 signaling may represent a novel pathway for promoting muscle regeneration and repair [40]. Meanwhile, the JAK1/STAT1/STAT3 pathway repressed MyoD1 and MEF2 expression, while the JAK2/STAT2/STAT3 pathway enhanced their expression, consistent with their opposite action on myogenesis. These studies indicate that different members of the JAK/STAT family differentially regulate muscle cell proliferation and differentiation, but it is not well known which ligands engage each specific effect [13].

On the other hand, the SOCS family of proteins is involved in regulations of JAK/STAT signaling [42]. SOCS1 and SOCS3 target JAK1 and gp130, respectively, and prevent cytoplasmic STATs from being activated, in a feedback inhibitory mechanism that regulates this pathway during myogenesis [13]. It has been observed in human myoblast that SOCS3 overexpression resulted in an increased expression of genes associated with skeletal muscle growth, and SOCS3 signaling during aging is dysregulated [13]. Moreover, studies performed in transgenic mice where IL-6 is overexpressed suggest the involvement of SOCS3 in muscle atrophy [43]. Bonetto et al., demonstrated in a murine model of cachexia that SOCS3 was unchanged or even reduced in the more atrophic muscles when compared with control muscles [44].

In our model, we observed that e-RA did not change pSTAT1 nor SOCS1, but decreased pSTAT3 and SOCS3 protein levels when compared with healthy animals. TOFA treatment did not modulate pSTAT1/SOCS1, but tended to decrease pSTAT3 and SOCS3 when compared with e-RA rabbits. These data together with the restoration in MyoD1 levels, the tendency to restore Myogenin, and the unaltered PAX7 suggest that neither cell proliferation nor differentiation alterations induced by arthritis were reverted by TOFA, despite a restoration in muscle mass [32,33].

The modulation of pSTAT1/SOCS1 and pSTAT3/SOCS3 in synovium and muscle is different. We have previously reported that TOFA remarkably decreased pSTAT1 and SOCS1 levels, keeping pSTAT3 and SOCS3 increased in the synovium in our e-RA model [45]. The effects of JAK/STAT pathway modulation can vary depending on the tissue and cell type involved. Therefore, understanding the downstream elements and the specific signaling pathways involved is crucial when studying the effects of inflammation and its blockade on muscle homeostasis. This knowledge can also help in the development of more targeted therapeutic approaches for the treatment of muscle diseases.

Different data support the key role of the JAK/STAT pathway in muscle differentiation [46]. Therefore, the long-term effect of TOFA in muscle mass and function in RA patients needs to be further studied. It is important to note that treatment of inflammation and disease activity may not completely reverse the muscle deterioration associated with sarcopenia. A recent study has revealed that, in RA patients on different disease-modifying antirheumatic drug (DMARD) regimens using the Treat-to-Target (T2T) strategy, the tight control of disease activity failed to improve long-term body composition or muscle function [7]. In our model, although no changes in PAX7 were observed for any group, TOFA treatment restored MyoD1, muscle mass, CSA, CSD, type II fibers, and the myonuclei number, and tended to restore Myogenin. These changes may indicate an upregulation in myoblast differentiation and muscular regeneration as an early event after TOFA treatment [32,33].

To date, no data exist on the effect of TOFA on muscular improvement in RA patients. A systematic review performed by Hein et al., did not show a significant change in appendicular lean mass delta with any DMARD, including TOFA [47]. Recently, the RAMUS Study, an observational, single-arm, experimental medicine study designed to test the effect of TOFA in muscle mass, has not rendered any data [48]. Therefore, the careful monitoring of patients and further research is needed to fully understand the effects of JAK/STAT inhibition on muscle homeostasis in the long term.

Our model also reflects the association between JAK inhibitors and increased CK in patients with inflammatory disorders [22,23,25]. Our rabbit treated with TOFA had increased CK expression in gastrocnemius associated with a concomitant decrease in creatine in muscle, although no changes in serum CK were observed. Our results are in line with published data where CK activity was associated with muscle weakness in RA patients [49]. Moreover, a decrease in CK, alanine aminotransferase, or aspartate aminotransferases, all muscle markers, was associated with reduced muscle mass and strength in these patients [50,51]. Serum CK increases in several acute inflammatory conditions, such as destructive myopathies, as a consequence of muscle injury [52]. Intriguingly, serum CK levels and activity correlated inversely with the inflammatory status in RA [53]. In fact, serum CK levels have previously been proposed as an inflammatory marker in RA patients. Different trials described that serum CK increased within the first two weeks of TOFA therapy, and plateaued from 8 weeks to 6 months after treatment, then remaining within the normal range, and was not associated with clinical myopathy [22,25]. In our model, after 2 weeks of TOFA treatment, we observed a tissue-specific CK increase with unchanged serum CK, which may indicate some specific muscle mechanisms modified by this drug. It is plausible that this increase in muscle CK has a different dynamic than serum CK, probably indicating that the increase in muscle CK must reach high levels before being released to serum and being able to detect the increase in this fluid.

Therefore, the increase in muscular CK, together with an increase in muscle mass, supports the role of this enzyme as a valuable marker of muscle gain following treatment with JAK inhibitors.

In conclusion, our data provide novel insights into the effects of JAK inhibitors in muscle remodeling during RS. However, the disorganization of muscle fiber myonuclei and the partial restoring to baseline of the muscle cell differentiation markers in the tissue raise the question of whether these treatments could trigger a complete regeneration of muscle fiber cells or just induce the hypertrophy of cells already present. Consequently, there is a need for new pharmacological treatments that may strengthen muscle and be able to increase muscle hypertrophy, being more important in patients with an incorrect articular response.

## 4. Materials and Methods

### 4.1. Experimental Model of Arthritis in Rabbits

Animal handling and experimentation complied with the Spanish Regulations and the EU Guidelines for the Care and Use of Laboratory Animals and were approved by the Institutional Review Board for Research of the Jiménez Díaz Foundation University Hospital and Research Institute (PROEX 19/019).

New Zealand white male rabbits, three months old, 2.5–3 kg weight (Granja San Bernardo, Navarra, Spain), were housed individually in cages exposed to a 12 h light/dark cycle and maintained on an ad libitum diet of food and water. After two weeks of adaptation to our facilities, rabbits were randomly assigned into three groups: healthy (*n* = 7), antigen-induced arthritis (experimental RA (e-RA), *n* = 7), or e-RA rabbits treated with TOFA (e-RA+TOFA, *n* = 7). Body weight was assessed weekly. e-RA was carried out in rabbits as described [16,45] (Figure 1A). Briefly, a solution of 4 mg/mL ovalbumin (OVA) (Sigma-Aldrich, St Louis, MO, USA) in saline (0.9% NaCl) was emulsified with an equal volume of Complete Freund’s Adjuvant (Difco, Detroit, MI, USA). Rabbits were immunized against OVA by two 1 mL intradermal injections, spaced 15 days apart. Five days after the second injection, 1 mL of OVA (5 mg mL^−1^ in saline) was injected intraarticularly into both knee joints on a weekly basis over the following four weeks (Figure 1A). Healthy controls received weekly intra-articular saline injections. Gelatin capsules of 10 mg/kg/day TOFA or placebo were orally administered via gavage at the time of the second intra-articular injection and continued until euthanization, on day 28 after challenge (Figure 1A) [45]. In order to explore the initial pharmacological effects of tofacitinib at the peak of inflammation, the last dose of this JAK inhibitor was given one day after the fourth intra-articular injection and four hours before rabbits were euthanized.

### 4.2. Tissue Collection

After overnight fasting, rabbits were bled from their marginal ear vein and euthanized with an overdose of intracardiac sodium thiopental (50 mg/kg; Thiobarbital, Braun Medical SA, Barcelona, Spain). Then, 10 mL of blood was allowed to clot and serum was collected, aliquoted, and stored at −80 °C. Both limbs were shaved and an anterior skin incision was made from patella to mid-paw to dissect TA and EDL and proximal gastrocnemii from both limbs as described [16]. TA muscles were transferred to a cell plate for anterior/posterior photographs. A 1 mm segment of the TA mid-belly, defined as the widest identifiable region, was sectioned, dehydrated in talc powder, placed cross sectionally in OCT (Fisher Scientific, Waltham, MA, USA), and stored at −80 °C. EDL muscles were pooled and transferred to 0.2% type I collagenase (Sigma-Aldrich) in Dulbecco’s Modified Eagle’s Medium (DMEM, Lonza, Basel, Switzerland), 4.5 g/L glucose, and 1% L-glutamine with 110 mg/mL-sodium pyruvate, and incubated at 37 °C for 1 h. Under a stereoscopic microscope, EDL muscle was flushed in warm medium until fibers were released as described [54]. Gastrocnemii muscles were snap frozen in liquid nitrogen and stored at −80 °C.

### 4.3. Determination of Serum Biochemical Markers

Serum levels of CRP and myostatin were measured by ELISA kit (Abcam, Cambridge, UK, and Cloud Clone Corp., CEB653, Houston, TX, USA) following the manufacturer’s instructions [16].

### 4.4. Histological Studies

Two consecutive 5 μm TA muscles sections were oriented in the same plane and attached to a single microscope slide (Superfrost PLUS; Fisher Scientific). H&E stained TA cross sections were photographed using an automated iScan Coreo slide scanner (Ventana Medical Systems, Tucson, AZ, USA) with 200× magnification. The cross-sectional diameter (CSD) and cross-sectional area (CSA) were measured using the Image J software package 1.49v (NIH, Bethesda, MD, USA). CSD was measured using the Ferret’s diameter, defined as the maximum diameter across the lesser aspect of the section. Parallel horizontal lines at the top and bottom of the image ensured consistent perpendicular measurements. CSA was calculated after tracing the outline of the cross section. All measurements were taken manually and blinded to each group.

ATPase staining was performed in TA cross sections as described [16]. Briefly, cross sections were incubated for 45 min in 0.1 M glycine/NaCl buffer pH 9.4 with 0.75 M CaCl_2_ and 5 mg ATP (Sigma-Aldrich). After being rinsed in distilled water, sections were incubated in 2% cobalt chloride and then immersed in dilute (1/10) ammonium chloride to develop type 1 (white) and type 2 (black) fibers. After dehydration, sections were mounted with DPX mounting medium (VWR International Ltd., Lutterworth, UK).

### 4.5. Fluorescent Labeling of EDL Fibers

Ten fiber segments from each animal were collected in a plate covered with 10% Matrigel (Corning, New York, NY, USA) forming three different pools: healthy, e-RA, and e-RA+TOFA. From each pool, fibers were transferred to a chamber slide and fixed in 10% formalin, rinsed, and permeabilized with 0.5% triton. Fibers were incubated in 100 nM rhodamine-phalloidin (Cytoskeleton, Denver, CO, USA) for 45 min to stain actin. DAPI (1 μg/mL, Invitrogen, Eugene, OR, USA) was incubated for 30 min to counterstain nuclei. Slides were mounted in Fluorsave (Calbiochem, Merck Millipore, Darmstadt, Germany) and visualized with a Leica TCS SP2 instrument. Myonuclear count was performed using Image J software package. Myonuclei number was assessed in myofiber segments of 100 μm of length and is expressed as number of nuclei per µm^2^ of fiber segment.

### 4.6. RNA Isolation and RT-PCR

Total RNA was isolated from gastrocnemius using TRIzol reagent (Roche Diagnostics, Barcelona, Spain) following the manufacturer’s instructions. A total of 1 µg RNA was reverse transcribed with a High Capacity cDNA Reverse Transcription Kit (Applied Biosystems, San Francisco, CA, USA) at 2.5 U/μL, including the following reagents, in the same reaction: RNase Inhibitor 1 U/μL, Random Hexamers 2.5 U/μL, MgCl_2_ 5 mM, PCR buffer II 1×, and dNTPs 1 mM. RNA expression was quantified by single-reporter real-time PCR using the Step One Plus Detection system (Applied Biosystems). Commercial TaqMan^®^ primers and probes were used for MCP-1, MSTN, IL-1, IL-6, and TNFα, as well as the endogenous controls glyceraldehyde-3-phosphate dehydrogenase (GAPDH) and eukaryotic 18S ribosomal subunit (18S). SYBR Green^®^ primers were designed as previously described [16] and used to measure MuRF-1, atrogin-1, and the endogenous control peptidylprolyl isomerase A (PPIA). Gene expression levels were calculated with the ΔΔCt method.

### 4.7. Western Blot

Frozen gastrocnemius was crushed using a mortar and a pestle cooled with liquid nitrogen. Then, 50 mg of pulverized tissue was homogenized in a buffer containing 15 mM HEPES, 10% glycerol, 0.5% NP-40, 250 mM NaCl, 1 mM EDTA, 1 mM phenylmethanesulfonylfluoride (PMSF), and a phosphatase- and protease-inhibitor cocktail (Sigma-Aldrich). Protein concentration was determined by the BCA. Then, 40 μg of protein was subjected to 10–15% SDS-PAGE and transferred to nitrocellulose membranes. After blocking in 3% skimmed milk (1 h, RT), membranes were incubated overnight at 4 °C with anti-myostatin/GDF8 antibody (0.4 μg/mL; R&D Systems, AF788, Minneapolis, MN, USA), pSTAT3 (2.5 μg/mL; R&D Systems, MAB4607), pSTAT1 (5 μg/mL; Affymetrix eBioscience, 14-9008, San Diego, CA, USA), SOCS1 (1 μg/mL; Abcam, ab9870, Cambridge, UK), SOCS3 (4 μg/mL; Abcam, ab14939), Pax7 (2.8 μg/mL, Developmental Studies Hybridoma Bank, AB 528428, Iowa City, IA, USA), Myogenin (2 μg/mL; Abcam, ab82843), and MyoD1 (1 μg/mL; Abcam, ab16148). Antibody binding was detected with chemiluminescence using secondary antibodies: anti-goat-HRP 1:5000 (Merck Millipore), anti-rat-HRP 1:20,000 (Fisher Scientific) or anti-mouse-HRP 1:5000 (GE Healthcare LifeSciences, Piscataway, NJ, USA. α-tubulin protein levels were employed as the loading control.

### 4.8. Creatine Kinase, Creatine, and Pyruvate Analysis

Creatine kinase in serum was analyzed in the Department of Clinical Analysis and Biochemistry at Hospital Fundación Jimenez Díaz following standard protocols.

Creatine concentration was determined using a creatine assay kit and following the manufacturer’s recommendations (Sigma-Aldrich), and colorimetric reaction was measured at 570 nm in a microplate reader (Tecan Infinite, Tecan Iberica Instrumentacion S.L, Barcelona, Spain).

Pyruvate was measured using a pyruvate assay kit (Abcam, ab65342) following recommendations and measured in a microplate reader at 570 nm (Tecan Infinite).

### 4.9. Statistical Analysis

Distribution of the variables was assessed using Shapiro–Wilk and Levene’s tests. In view of the lack of normality and homogeneity drawn by these tests and considering the small sample size, non-parametric tests were chosen for the analysis. Kruskal–Wallis test and Dunn’s post hoc test were run for multiple group comparisons, while two-group comparisons were performed with the Mann–Whitney U test for independent samples, respectively. A *p*-value of less than 0.05 was considered significant. Statistical analysis was performed using Windows SPSS 21.0 (SPSS, Inc., Chicago, IL, USA).

## Data Availability

The data that support the findings of this study are available from the corresponding author upon reasonable request.
